# Case-oriented selection of investigation methods in direct access: A comparison between physiotherapy trainees at professional colleges and in bachelor's study courses

**DOI:** 10.3205/zma001157

**Published:** 2018-02-15

**Authors:** Ralf Konrad, Max Geraedts

**Affiliations:** 1 Private Universität Witten/Herdecke, Fakultät für Gesundheit, Institut für Gesundheitssystemforschung, Witten, Germany; 2 Philipps-Universität Marburg, Fachbereich Medizin, Institut für Versorgungsforschung und Klinische Epidemiologie, Marburg, Germany

**Keywords:** physical therapy speciality, education, diagnosis, Drect access

## Abstract

**Objective: **Direct access to physiotherapy services is currently discussed in Germany. Its introduction would mean that initial diagnoses must be made in physiotherapy practices as well. However, it was not yet investigated whether the current training in physiotherapy is sufficient for this, and whether there are differences between the training systems. This study aims to answer the question of whether trainees at the end of Bachelor’s studies (BS) are more reliably able to assess the case-related suitability of examination methods than professional college students (FS).

**Methodology: **Questionnaires were developed to assess the suitability of examination methods for diagnostic inquiries. All professional colleges and bachelor’s study courses listed with the German Physiotherapy Association were asked to present the questionnaires to their final classes.

**Results:** In 216 addressed professional colleges and 24 bachelor’s study courses, the return rate was 9.26% for professional colleges and 33.33% for study courses. One hundred thirty-eight questionnaires from students in 8 study courses and 368 questionnaires from students at 20 professional colleges were evaluated. The mean of correct decisions in total (of max. 54) was 19.01 (BS) or 15.73 (FS); in structure-related and function-related examination methods (of max. 42), it was 17.22 (BS) and 14.8 (FS); in activity-related methods (of max. 12), it was 1.97 (BS) and 0.89 (FS).

Out of a max. of 49 examination methods, 23.45 (BS) and 26.72 (FS) were stated as unknown.

**Conclusion:** The university students made correct decisions on the suitability of examination methods significantly more frequently than the professional college students. However, the determined group difference is low.

Overall, the results do not appear sufficient for direct access. Training would have to be adapted for this purpose.

## Zusammenfassung

**Zielsetzung: **Derzeit wird in Deutschland der Direktzugang zu physiotherapeutischen Leistungen diskutiert. Dessen Einführung würde bedeuten, dass auch in physiotherapeutischen Praxen Erstdiagnosen gestellt werden müssen. Ob die gegenwärtige Ausbildung in der Physiotherapie dafür ausreicht und ob es dabei Unterschiede zwischen den Ausbildungssystemen gibt, wurde bisher nicht untersucht. In der vorliegenden Studie soll die Frage beantwortet werden, ob Auszubildende am Ende eines Bachelorstudiums (BS) die fallbezogene Eignung von Untersuchungsmethoden zuverlässiger einschätzen können als Fachschüler (FS).

**Methodik: **Es wurden Fragebögen entwickelt, in denen die Eignung von Untersuchungsmethoden für diagnostische Fragestellungen beurteilt werden sollte. Alle beim Deutschen Verband für Physiotherapie gelisteten Fachschulen und Bachelorstudiengänge wurden gebeten, die Fragebögen ihren Abschlussklassen vorzulegen.

**Ergebnisse: **Bei 216 angeschriebenen Fachschulen und 24 Bachelorstudiengängen lag die Rücklaufquote für Fachschulen bei 9,26% und für Studiengänge bei 33,33%. Es wurden 138 Fragebögen von Studierenden aus 8 Studiengängen und 368 Fragebögen von Schülerinnen und Schülern aus 20 Fachschulen ausgewertet. Die Mittelwerte richtiger Entscheidungen insgesamt (von max. 54) lagen bei 19,01(BS) bzw. 15,73(FS), bei struktur- und funktionsbezogenen Untersuchungsmethoden (von max. 42) bei 17,22 (BS) und 14,8 (FS), bei aktivitätsbezogenen Methoden (von max. 12) bei 1,97 (BS) und 0,89 (FS).

Von max. 49 Untersuchungsmethoden wurden 23,45 (BS) und 26,72 (FS) als unbekannt angegeben.

**Schlussfolgerung:** Die Hochschüler trafen signifikant häufiger korrekte Entscheidungen zur Eignung der Untersuchungsmethoden als die Fachschüler. Der ermittelte Gruppenunterschied ist aber gering.

Insgesamt erscheinen die Ergebnisse für den Direktzugang als nicht ausreichend. Die Ausbildung müsste zu diesem Zweck angepasst werden.

## 1. Introduction

### 1.1. Background

According to the Statistics Yearbook of 2012, Germany has the oldest population in Europe, and the second oldest in the world after Japan [1]. The population projection of the Statistics Yearbook of 2016 predicts that the age quotient will increase from 35 in the year 2014 to 49 or 50 (depending on the degree of immigration) [2]. In this aging society, growing demands on health and long-term care of the population are expected. Between 2004 and 2014, the number of outpatient treatments in Germany increased by 152 million [3]. The number of physicians did not increase to the same degree in this period. At the same time, the weekly work hours provided by physicians is decreasing. The average age of physicians is also rising [3]. If this development continues, gaps in medical care may form. 

In this situation, it is being controversially discussed for the field of physiotherapy whether substitution or delegation of physicians’ tasks can solve the problem. Specifically, there is the question of whether shifting physicians’ tasks in this manner would place patients at risk [4], [5], [6], [7], [8], [9], [10]. Two models for taking over physicians’ tasks are currently discussed for physiotherapy:

In a first variant, therapists are to make independent decisions regarding the type, frequency and number of treatments after a physician’s referral. The draft law on the Medicinal Products and Aids Supply Law which was decided by the Federal Cabinet on 31 August 2016 initiates model projects for this so called “unspecified referral” [11]. Two model projects which have already been in progress since 2011 are showing the first positive results [12], [13].

The second feasible model would be direct access to physiotherapy services without first contacting a physician. This is already a reality via the detour of the Non-Medical Practitioners Act. In a judgement with a guideline dated 26 August 2009, the Bundesverwaltungsgericht (BVerwG) [Federal Administrative Court] notes that physiotherapists can be granted a non-medical practitioners’ license which is limited to the field of physiotherapy. However, this regulation does not include patient care at the expense of statutory health insurance funds.

Within the scope of a survey by Bury and Stokes from the year 2013, 58% (with a return rate of 68%) of the member organisations of the World Confederation for Physical Therapy (WCPT) stated that direct access to physiotherapy is permissible in their states [14]. According to the Direct Access Report of the WCPT of January 2013, direct access is legally permitted in the following European states: Denmark, Finland, Hungary, Ireland, Liechtenstein, Lithuania, Malta, the Netherlands, Norway, Poland, Portugal, Spain, Sweden and Great Britain [15]. There are major differences as to whether and to what extent public cost carriers cover or refund direct access treatment costs. According to information provided by the WCPT, there is no unrestricted direct access in Croatia, the Czech Republic, France, Italy, Luxembourg, Romania, Slovenia and Switzerland. Only self-payers are able to directly access physiotherapy services. In a systematic review from the year 2015, Ohja et al. concluded that direct access patient care has the potential to save costs and improve patient-related outcomes for musculoskeletal complaints. There were no indicators of lower patient safety [16]. However, these experiences are not readily transferable to the German healthcare system. Unlike the academic training in direct access states, physiotherapy training in Germany primarily takes place at professional colleges. The university-based education rate of 10-20% recommended by the science council [17] is opposed by an estimated quota of 4% [18]. Since 3.10.2009 until 31.12.2017, model study courses are being implemented for this purpose. The evaluation of the Bundesministerium für Gesundheit (BMG) [Federal Ministry of Health] to the German Bundestag of August 2016 [19] evaluated the results of these model study courses as positive as a tendency. On 01 December 2016, the German Bundestag decided on an extension of 4 years regarding this matter [20]. 

The statutory basis of physiotherapy training is the Ausbildungs- und Prüfungsverordnung für Physiotherapeuten (PhysTh-APrV) [Training and Examination Regulation for Physiotherapists] from the year 1994 [21]. Some federation states and the Deutscher Verband für Physiotherapie (ZVK) [German Physiotherapy Association] have also published their own Curricula [22], [23], [24], [25], [26], [27], [28]. The content design of bachelor’s study courses is specified in the module handbooks of the universities. The guarantee of minimum content standards in the subject is verified within the scope of accrediting the study courses, in accordance with the decision by the education ministers’ conference of 03.12.1998 [29]. A systematic content verification of these two training routes compared to the training guideline of the WCPT [30] showed significantly greater deficits of professional college education overall [31], [32]. Specifically, the scientific basis of therapeutic actions and lifelong learning is nearly omitted in the PhysTh-APrV. In the module books of the bachelor’s study courses, the specifications of the guideline are nearly completely fulfilled for this field. Compared to the current German system, a significant difference in direct access patient care consists of the task of providing an initial diagnosis. In order to ensure safe patient care, the presence of indicators of serious specific pathologys (red flags) must first be verified [33]. In 2010, Beyerlein determined clear uncertainties among German physiotherapists in this regard [34]. If red flags are present, medical clarification is mandatory, since the determination of the diagnosis is outside the limits of physiotherapeutic expertise. 

In cases without red flags, physiotherapists should be able to make the diagnosis. It can be presumed that general techniques for obtaining findings are taught in German physiotherapy training. However, these methods are only suitable for determining a general status; they are generally not sufficient for drawing definitive conclusions regarding the underlying pathology. Common musculoskeletal examination concepts according to the methods of Cyriax [35], [36] or Brügger [37] are questionable or refuted regarding their evidence [38], [39], [40], [41], [42]. Classifications such as those described by McKenzie [43], [44] or Waddell [45] aim at a treatment oriented classification into categories. A physiotherapeutic diagnosis at all levels of the International Classification of Function, Disability and Health (ICF) [46] is not possible with such systems.

Meanwhile, however, there are a large variety of examination methods for all levels of the ICF, whose evidence has been scientifically verified. In order to increase the significance of clinical tests, many authors recommend combining several tests into a cluster [47], [48], [49], [50], [51]. The knowledge of such tests and the ability to appropriately select and apply them is required by the minimum professional standards for physiotherapists in states with direct access [30], [52], [53], [54], [55]. 

The degree to which students from professional colleges or bachelor’s study courses in Germany have such knowledge or differ in it was not investigated thus far. Clarifying this is significant for occupational policy decisions, and constitutes a first step in further developing physiotherapy training with an orientation towards future tasks.

#### 1.2. Objective

In view of this background, the study aims to verify to what extent German physiotherapy training lays the groundwork for the ability to select examination methods for producing a diagnosis. Herein professional college students are compared to bachelor’s students. 

#### 1.3. Study Question

At the end of their training, are bachelor’s students able to assess the case-related suitability of examination methods more reliably than professional college students are?

#### 1.4. Hypotheses

Null hypothesis: Academically trained physiotherapists do not assess the suitability of examination techniques correctly more often than non-academically trained physiotherapists do.

Alternative hypothesis: Academically trained physiotherapists assess the suitability of examination techniques correctly more often than non-academically trained physiotherapists do.

## 2. Method

### 2.1. Development of the questionnaires

In order to investigate the study question, questionnaires with case vignettes were developed. For this purpose, the example of the Netherlands was used to research some symptom combinations that were treated in direct access with particular frequency [56]. For these symptom combinations, clinical practice guidelines were searched using the guideline search function of the Arbeitsgemeinschaft der Wissenschaftlichen Medizinischen Fachgesellschaften e.V. (AWMF) [Association of the Scientific Medical Societies, reg. assoc.] [57], in the Physiotherapy Evidence Database (PEDro) [58], in the Guidelines International Network [59] and with the Pubmed search engine.

Using the guidelines shown in table 1 (Tab. 1), typical signs and symptoms were taken over for the case description. Using the guideline recommendations, suitable examination methods were determined. For the case vignette “shoulder complaints”, the Clinical Reasoning Algorithm of Cools [60] was additionally applied.

“Incorrect” examination methods for the individual case vignettes were researched in the guidelines, textbooks [61], [62], [63] and online tutorials [64], [65], [66]. A total of 37 examination methods with structure-related and function-related outcomes and 12 methods with primarily activity-related outcomes were selected. 

Personal- and environment-related contextual factors were randomly allocated to the case vignettes, insofar as possible. A total of five case vignettes with the following symptoms were developed: Frozen Shoulder (adhesive capsulitis), non-specific low back pain, low back pain with radicular pain, knee pain and hip pain. For these case vignettes, eight questions were asked; herein, participants were provided with a selection of suitable and unsuitable examination methods. For each examination method, participants had to choose “correct”, “wrong” or “not known”. Table 2 (Tab. 2) shows the examination methods to be evaluated on the questionnaire. 

The procedure described by Lawshe [67] was selected to verify the content validity of the questionnaire. The main reason for this was that the symptoms for the case descriptions and the examination procedures that were evaluated as correct were specified by the guidelines, and did not have to be determined using a more complicated procedure, e.g. according to Moore Benbasat [68]. 

For this purpose, the questionnaire was presented to six experts for assessment. 

The experts were selected according to the following criteria:

- University diploma in physiotherapy
- Several years of occupational experience
- Teaching activity in professional college and university programmes in the field of musculoskeletal examination techniques
- Teaching activity in a program in which direct access is taught
- Very good German language skills

Six of the eight contacted experts were willing to carry out the task. 

Following this, a pilot was implemented with 17 students who had completed a professional college education and 17 students in bachelor’s physiotherapy study courses. The goal of this pilot was to verify the practicality of the questionnaire and obtain data for assessing the sample size. In the pilot, the maximum amount of time required to work on the questionnaire was 20 minutes. The participants were able to work on it without further comprehension questions regarding the case vignettes. Questions regarding the background and procedure of the investigation were clarified in advance. Acceptance was high. Subject to the prerequisite that apart from the informed consent, no personal data were collected, all those addressed were willing to participate and handed in the completed questionnaire. On average, it took four minutes to evaluate each questionnaire. 

In view of this background and with a Whole Test CVI of 0.73, the questionnaire was deemed suitable for answering the study question.

Based on the experiences made in the pilot, the information text for the participants was revised.

#### 2.2. Participant recruitment:

All training institutions in the directory of schools (N=216) [69] and all physiotherapy bachelor’s study courses in the list of study courses (N=24) [70] of the Deutscher Verband für Physiotherapie (ZVK) were contacted in writing and asked to participate. 

It is possible that educational institutions that only teach standardised diagnostic methods in their training to a lesser degree were less frequently willing to participate in the study. This potential selection bias was counteracted by agreeing that conclusions regarding the individual results of the schools cannot be drawn from the study results. The proportion of addressed trainees who were willing to participate was consistently very high. A selection bias in the direction of weaker trainees was therefore improbable.

#### 2.3. Inclusion and exclusion criteria

Inclusion criteria

- Physiotherapy students or bachelor’s students in a late phase of their training in which skills on examination of musculoskeletal disorders are no longer taught or prepared.
- Good skills in the German language, both written and spoken.
- Only university students who were studying in foundation or apprenticeship physiotherapy programmes were included.

Exclusion criteria:

- Additional started or completed training or further training in medicine.
- Additional started or completed occupational training in a related discipline (e.g. ergotherapy).
- For the group of professional college students: No students completing apprenticeship university studies in physiotherapy, therapy sciences or medical paedagogy.

#### 2.4. Implementation

The questionnaires were sent to the participating educational institutions via e-mail. They were instructed to ensure that trainees completed the questionnaires independently and without using aids. The time frame to work on the questionnaires was 45 minutes. The questionnaire included an information sheet with all required information for participants. Assurance was provided that the published data could not be used to draw conclusions regarding the results of individual participants or educational institutions. 

The participants confirmed with their name and signature on a separate list that they were participating on a voluntary basis. They were informed that beyond this, no personal data are collected. The completed questionnaires and signed participation confirmation sheets were returned via mail.

The data were evaluated using the SPSS program package. Normal distribution of data was verified using the Kolmogorow-Smirnov test and Shapiro-Wilk-Test. In order to test the hypothesis, the Mann-Whitney-U-test was implemented.

## 3. Results

### 3.1. Content validity of the questionnaire

The Lawshe procedure to verify the content validity of the questionnaire determined a Whole Test Content Validity Index (CVI) of 0.73.

#### 3.2. Sample size estimation

Based on the data from the pilot, the G-Power programme was used to determine a minimum sample size of *n*=63 participants/group. This calculation was based on a one-sided testing, 44.1% correct responses of university students and 22.1% correct answers of professional college students, α=0.05 and a power of 0.8. 

#### 3.3. Response rate

Out of the contacted 216 professional colleges and 24 bachelor’s study courses, 20 professional colleges (=10.64%) and five universities with eight study courses at differing locations (=33.33%) participated in the study. The average percentage of addressed trainees who were willing to participate amounted to 85% at professional colleges and 97% at universities. One participating professional college did not fulfil the inclusion criteria; trainees completed the questionnaires at home at two professional colleges. The results of these three educational institutions were not taken into account. The resultant response rates amount to 9.26% for professional colleges and 33.33% for study courses. A total of 138 questionnaires from university students and 368 questionnaires from professional college students were evaluated.

#### 3.4. Results of the statistical analysis

Since there was no normal distribution of data, the hypothesis testing was performed using the non-parametric Mann-Whitney-U-test.

Nearly all results of group comparisons were significant, with a significance level of α=0.05 (see table 3 (Tab. 3)). One exception consisted of the unsuitable examination methods (FR) which were erroneously classified as “correct” by the participants. Here, university students made 5.82 errors in the mean. Professional college students made this error 5.21 times on average.

Thirty seven different examination methods with structure- and function-related outcomes and 12 with primarily activity-related outcomes were provided for selection in the questionnaires. Participants were able to evaluate them as “correct”, “wrong” or “not known”. Table 4 shows the number of methods classified as “not known”. University students consistently used the classification of “not known” less often than college students did.

Overall, a correct/wrong decision had to be made regarding 54 examination methods (some of the 49 different examination methods were presented as possible selections in more than one question). Table 4 (Tab. 4) shows the number of correct right/wrong decisions in the comparison of education forms. Out of the 49 different methods, 22 are recommended for the respective diagnostic inquiry in the initially mentioned clinical guidelines. Out of these, 11.27 (51.23%, SD: 2.75) were recognised as “correct” by university students, and 10.15 (46.14%, SD: 2.7) were recognised as “correct” by college students.

**Figure 1:**
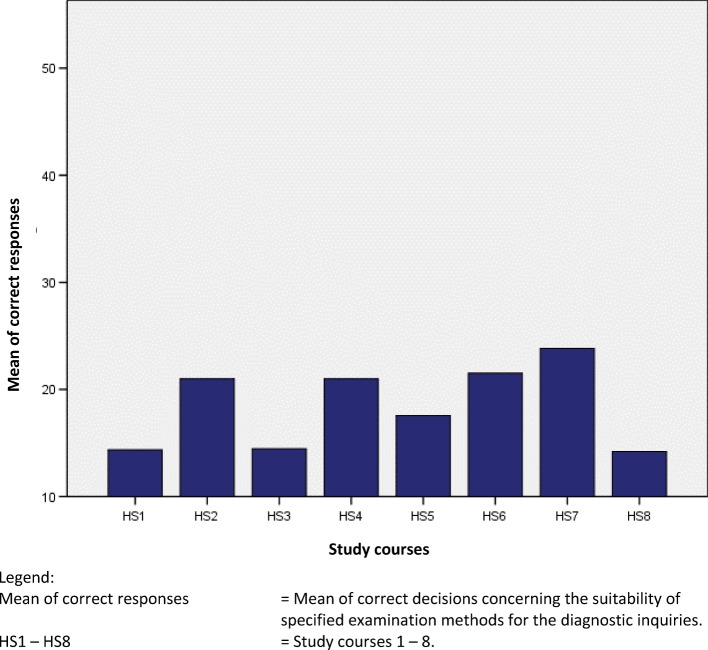
Comparison between study courses: Mean of correct decisions. (out of a total 54 possible)

**Figure 2:**
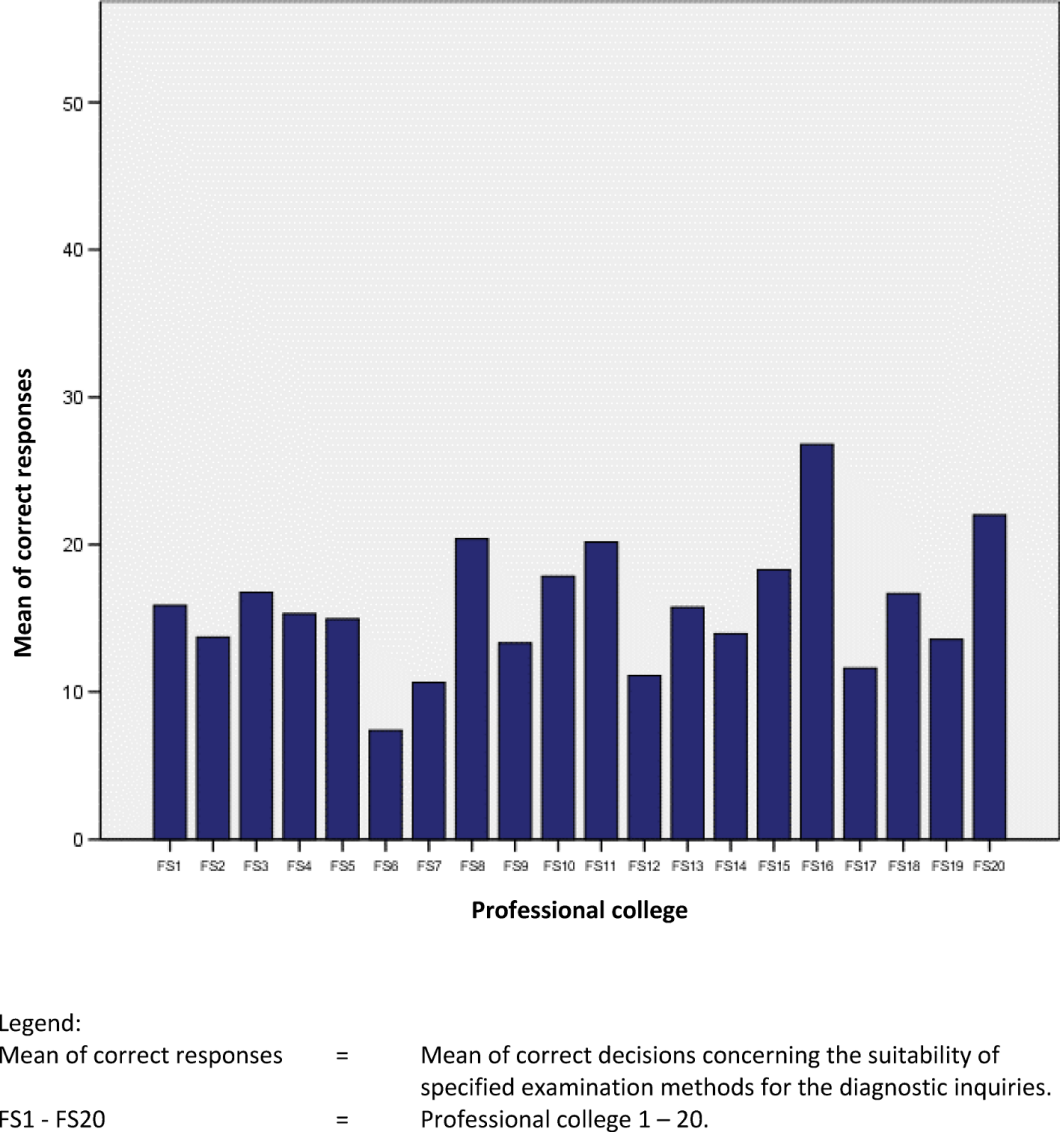
Comparison of professional colleges: Mean of correct decisions. (out of a total 54 possible)

The heterogeneity of German educational institutions regarding the investigated skill is shown in table 5 (Tab. 5), figure 1 (Fig. 1) and figure 2 (Fig. 2). The differences are particularly clarified by the wide ranges within the educational systems.

## 4. Discussion

### 4.1. Discussion of results:

In the comparison of training systems, a consistently significant advantage in favour of universities was shown regarding the investigated skill. However, the results appear unsatisfactory overall. 

In both groups, there is a high percentage of examination methods that are classified as “not known”. 41.36% of college students and 37.32% of university students do not know the examination methods recommended for commonly occurring symptoms by clinical guidelines. This percentage is noticeably higher among the examination techniques provided for selection overall. Evidently, examination techniques with activity-related outcomes in particular are not yet taught to a sufficient level in German physiotherapy training.

In order to make a diagnosis, it is especially important to choose the correct examination techniques. A quota of at least 60% correct responses can be expected for satisfactory results. In the arithmetic average, this result is not reached by professional college students (46%) nor by university students (51%). Regardless of whether physiotherapists should know suitable examination methods because they must make a diagnosis in the future as initial patient contacts or whether they must select suitable therapy forms within the scope of “unspecified referrals” using “clinical prediction rules”, or whether they must report the health status of patients at all levels of the ICF documentation, the results are unsatisfactory in both education routes. It must also be taken into account that the outcomes in scientific effectiveness studies are often measured using the utilised examination methods. Furthermore, knowledge of the examination methods is significant in order to understand corresponding journal articles. The high percentage of examination methods that were erroneously classified as “correct” is also alarming. In occupational practice, this can lead to wrong decisions in the Clinical Reasoning Process. Regarding the number of correct responses, it is also shown that examination methods with activity-related outcomes evidently do not play a major role in German physiotherapy education. In this field, professional college students only assessed 7.42% of methods correctly; university students, only 16.42%.

The range of correct answers shown in table 5 (Tab. 5) and the standard deviations show a high variance. This shows that there are major differences between the individual educational institutions regarding the study question. The variance is significantly higher between professional colleges than between university-based study courses. 

On the one hand, possible causes of the determined deficits in the investigated skill of adequately selecting examination methods include the age of the PhysTh-APrV, which cannot take the physiotherapeutic and didactic developments of the past 23 years into account, and which also does not define any skills as education goals. On the other hand, the qualification levels of teaching staff in professional college education are not uniformly specified [71]. Furthermore, physiotherapists do not make a diagnosis in their current role. They merely obtain function findings. Thus far, it was not necessary to add diagnostic skills for direct access to the fundamentals of training. This also applies to the module books of the bachelor’s study courses. 

#### 4.2. Limitations

One methodological disadvantage of the Lawshe procedure is that a high CVI can be calculated even if important indicators to measure the construct are absent [72]. This disadvantage had to be accepted. Regarding acceptance and the response quota, even alternative methods to develop the questionnaire would not have made it possible to take the entire bandwidth of the possible complaints and examination methods for direct access into account. 

The professional college results are from six schools in North Rhine-Westphalia, two in Rhineland-Palatinate, one in the Saar region, three in Bavaria, one in Hamburg, three in Baden-Württemberg, one in Schleswig-Holstein, one in Lower Saxony, one in Berlin and one in Hesse. Therefore, no data are available from the Eastern federation states. 

The response rates of 9.26% in professional colleges and 33.33% in study courses appear to be acceptable. Herein it must be taken into account that within the survey period from June to October 2016, final classes that corresponded to the inclusion criteria were not available at all institutions. 

## 5. Conclusions

A university education in physiotherapy offers a significant advantage regarding the investigated occupational skill compared to professional college education.

However, the results of this study lead to the conclusion that there are clear deficits in both forms of education in this regard. An adaptation of the training requirements appears to be necessary. 

## Data

Data for this article are available from the Dryad Digital Repository: http://dx.doi.org/10.5061/dryad.hn1rh [73]

## Competing interests

The authors declare that they have no competing interests. 

## Figures and Tables

**Table 1:**
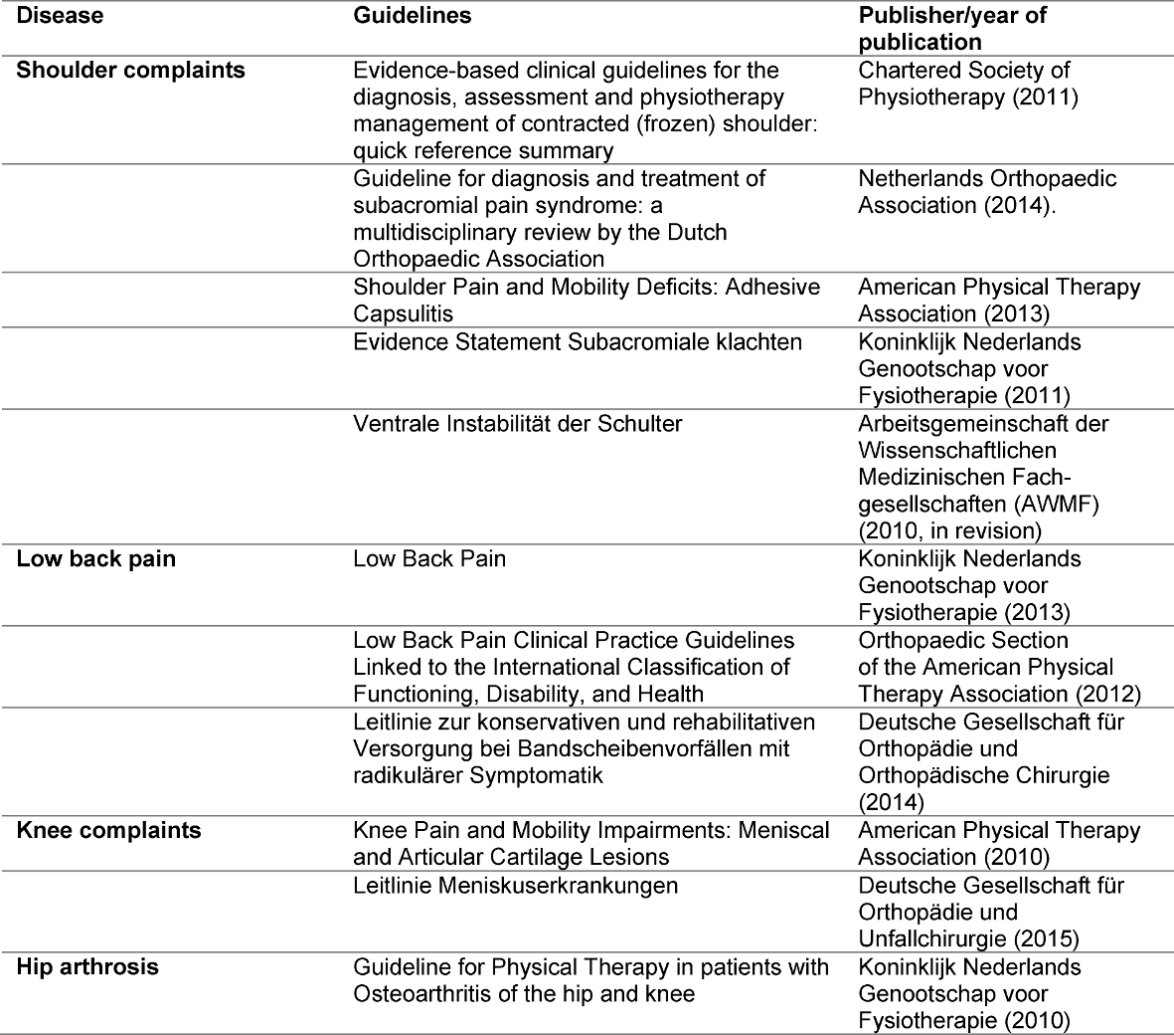
Utilised guideline

**Table 2:**
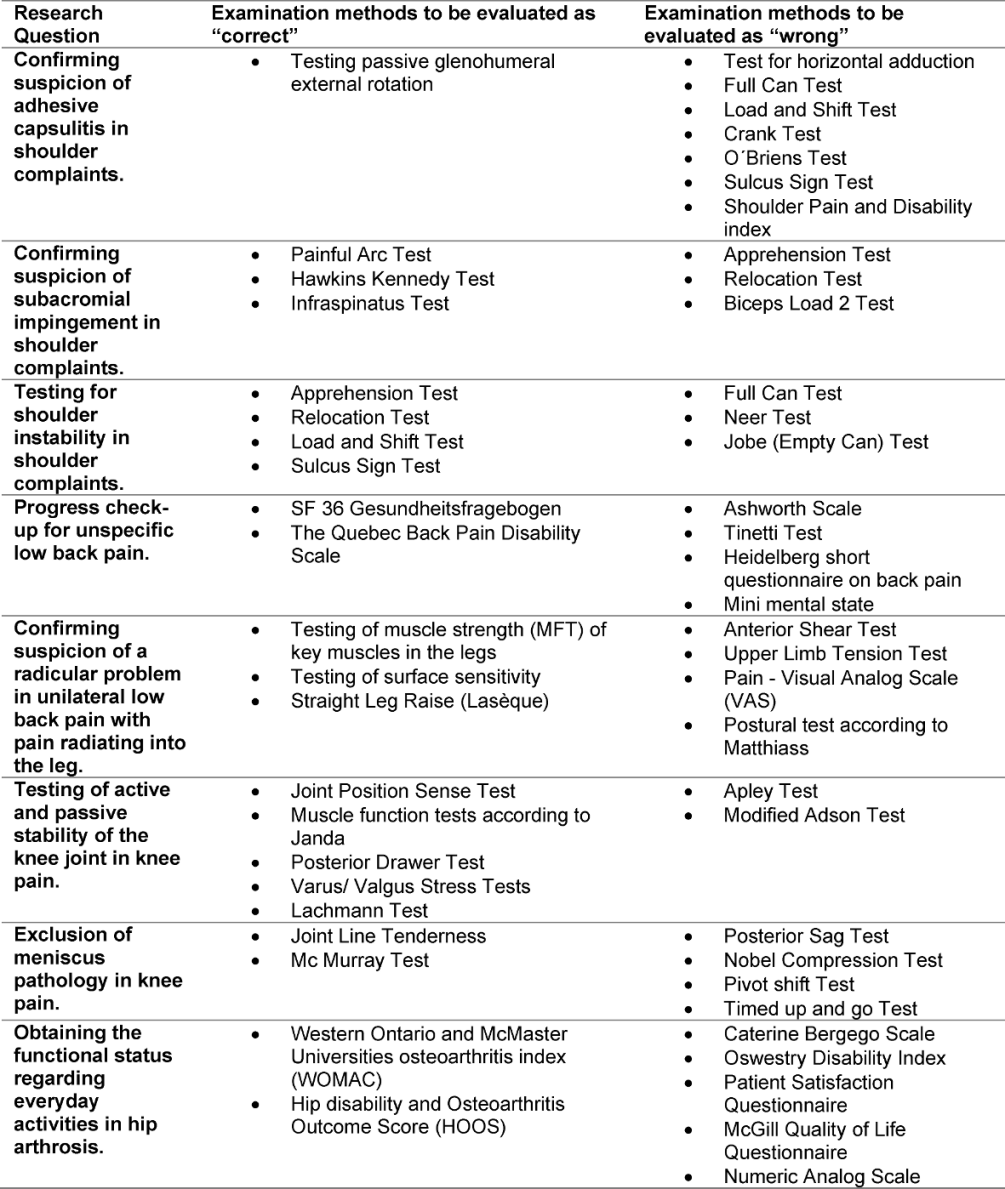
Examination methods

**Table 3:**
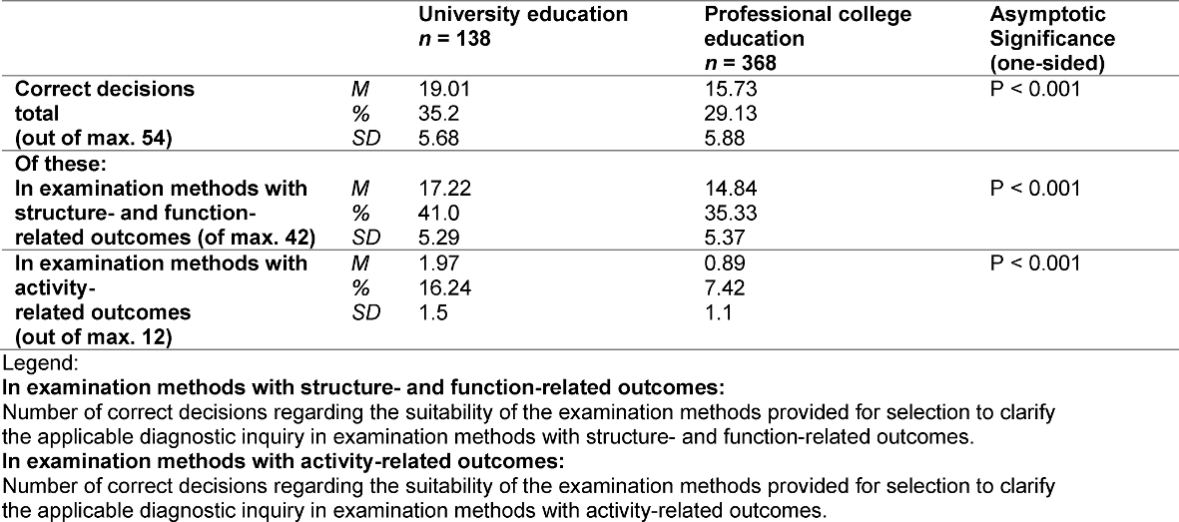
Share of correct decisions related to the total possible correct decisions

**Table 4:**
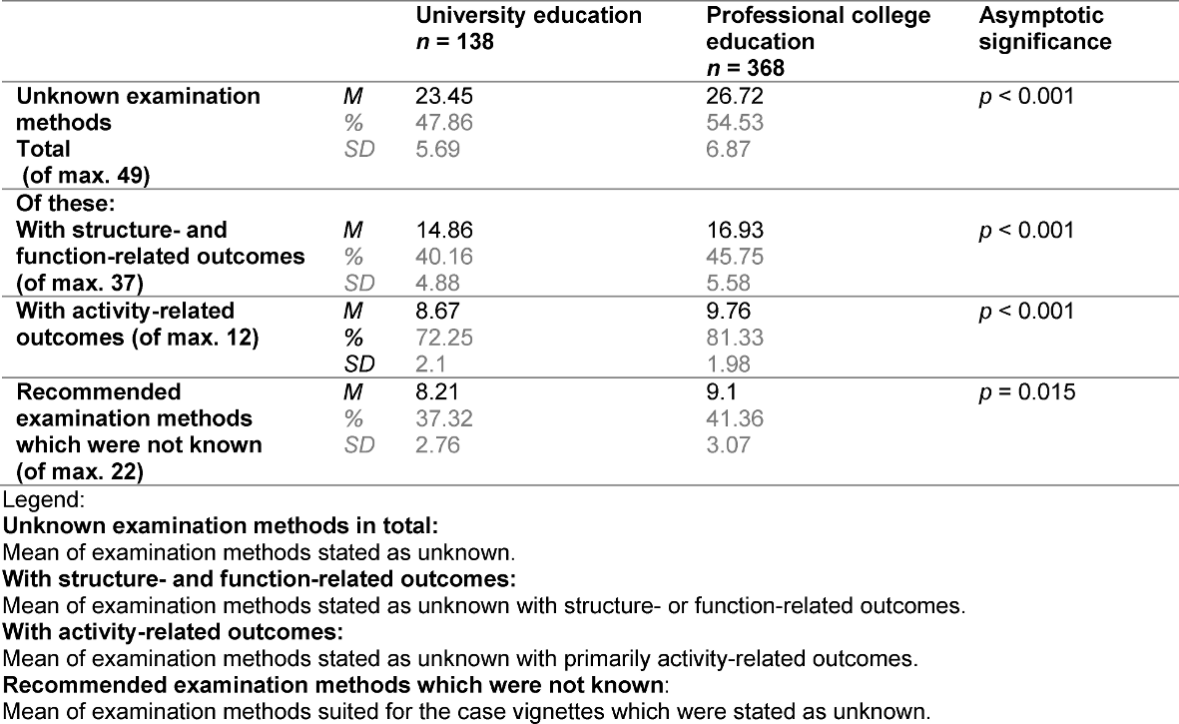
Examination methods stated as “unknown”

**Table 5:**
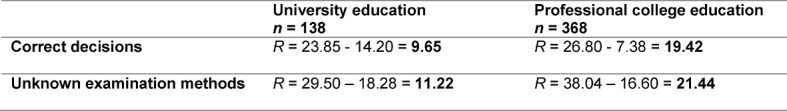
Comparison between educational pathways: Range of means
